# Sarcoid uveitis in a patient with multiple neurological lesions: a case report and review of the literature

**DOI:** 10.1186/s13256-018-1842-5

**Published:** 2018-10-23

**Authors:** Tomoko Ohno, Mami Ishihara, Etsuko Shibuya, Nobuhisa Mizuki

**Affiliations:** Department of Ophthalmology and Visual science, Yokohama City University School of Medicine, 3-9 Fukuura, Kanazawa-ku, Yokohama, Kanagawa 236-0004 Japan

**Keywords:** Neurosarcoidosis, Multiple cranial neuropathy, Brain parenchyma lesion, Sarcoid uveitis

## Abstract

**Background:**

Neurosarcoidosis is a rare complication, and cranial neuropathy is the most frequent manifestation of this disease. However, few cohesive reports have discussed multiple cranial neuropathies in Japanese patients with sarcoidosis. The present report discusses the case of a patient with sarcoid uveitis and multiple neurological findings. We further review relevant literature regarding Japanese patients with multiple cranial nerve palsies published within the past 34 years (from January 1982 to December 2016).

**Case presentation:**

We report findings associated with the case of a 56-year-old Japanese woman with granulomatous pan-uveitis who was later diagnosed as having sarcoidosis by skin and transbronchial lung biopsies. She presented right-sided Bell’s palsy and was treated with orally administered prednisolone. However, while prednisolone was tapered, she developed facial (VII) and vagus (X) nerve palsies, followed by brain parenchyma lesions, which were not associated with any additional neurological symptoms. Furthermore, she exhibited increased intraocular pressure in her right eye, and she underwent trabeculectomy. Our review of the literature revealed that 64 Japanese patients with sarcoidosis experienced multiple cranial nerve palsies between 1982 and 2016. The most commonly affected cranial nerves were the facial (VII) (73.4%) and glossopharyngeal/vagus (IX/X) nerves (48.4%). Palsies of two distinct cranial nerves were found in 40.6% of the patients, followed by palsies of three (23.4%) and four (18.8%) nerves. Almost all patients (98.3%) received systemic steroid therapy, and total or partial remission was achieved in almost all patients (96.5%).

**Conclusions:**

According to the literature, patients with multiple cranial nerve palsies associated with sarcoidosis respond well to orally administered steroid therapy. However, our findings suggest that careful follow-up is necessary for patients with neurosarcoidosis due to potential aggravation of neuropathy.

## Background

Occurring in approximately 3–15% of patients with sarcoidosis, neurosarcoidosis is a rare complication in which compression of neural tissue by granulomas or damage caused by infiltration/ischemia results in neurological symptoms [1]. Cranial neuropathy is the most frequent manifestation of neurosarcoidosis [2–5], and many patients present with two or more distinct cranial nerve palsies [2, 4]. Within the central nervous system (CNS), lesions of the lateral ventricle, hypothalamus, and pituitary gland are the most common [1]. We retrospectively discuss the case of a patient with sarcoidosis who presented with two distinct cranial palsies, multiple CNS lesions spanning the left temporal lobe and the subcortical white matter around both ventricles, in addition to ocular, skin, and lung lesions. As few cohesive reports have discussed multiple cranial neuropathies in Japanese patients with sarcoidosis, we further review the relevant published literature within the past 34 years (from January 1982 to December 2016).

## Case presentation

**Fig. 1:**
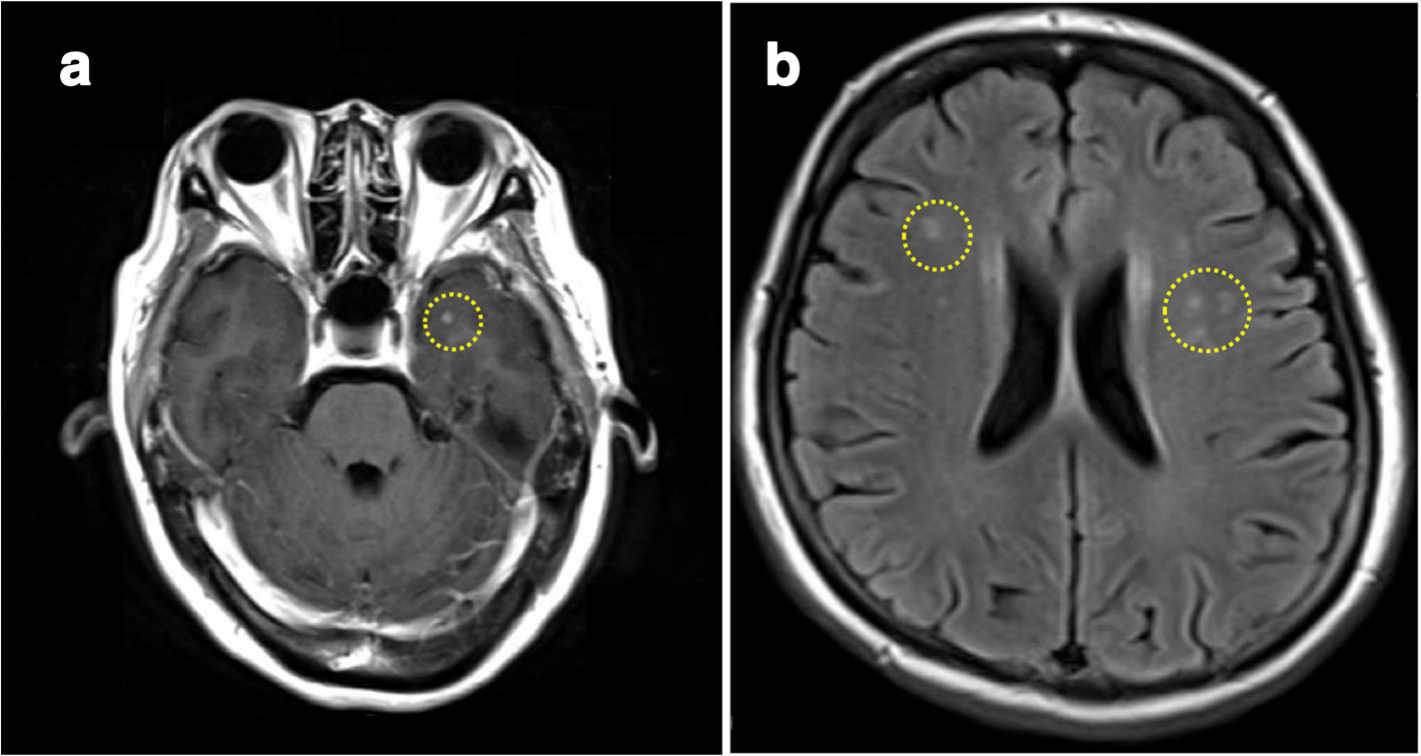
Gadolinium-enhanced magnetic resonance imaging scans of the brain. The images depict several small nodules in the left temporal lobe (**a**) and the subcortical white matter around both ventricles (**b**)

A 56-year-old Japanese woman presented with right-sided Bell’s palsy due to dysfunction of the facial nerve (VII) in October 2013. She had been diagnosed as having rheumatoid arthritis in August 2013 and treated with methotrexate orally. She had no relevant medical family history. Methotrexate was discontinued when she suffered from Bell’s palsy. Her condition improved following oral administration of 30 mg of prednisolone/day. Soon after prednisolone was discontinued in November 2013, methotrexate was resumed. In March 2014, she was admitted to a general hospital due to the appearance of bilateral floaters. Her best corrected visual acuity was 20/20 in her right eye and 20/25 in her left eye; an ophthalmological examination revealed bilateral granulomatous uveitis. Chest computed tomography (CT) revealed bilateral hilar lymphadenopathy and mediastinal lymph node swelling. Laboratory tests revealed elevated levels of serum angiotensin-converting enzyme (ACE) (35.0 IU/L; normal, 8.3–21.4 IU/L). She was histologically diagnosed as having sarcoidosis following skin and transbronchial lung biopsies. In February 2015, she developed dysphagia due to dysfunction of the vagus nerve (X), following which she was treated with 30 mg of prednisolone/day. Following an improvement in her symptoms, prednisolone dosage was tapered to 4 mg/day by August 2015. However, she experienced recurrence of nerve VII palsy in November 2015, despite continued treatment with 4 mg of prednisolone/day. In December 2015, magnetic resonance imaging (MRI) revealed brain parenchyma lesions (Fig. 1), although no neurological symptoms, such as motor/sensory paralysis or paresthesia, were observed. In January 2016, she experienced recurrence of nerve X palsy, following which she was treated again with 30 mg of prednisolone/day. In May 2016 (prednisolone, 20 mg/day), she exhibited increased intraocular pressure (IOP) in her right eye and was referred to our hospital for treatment.

**Fig. 2:**
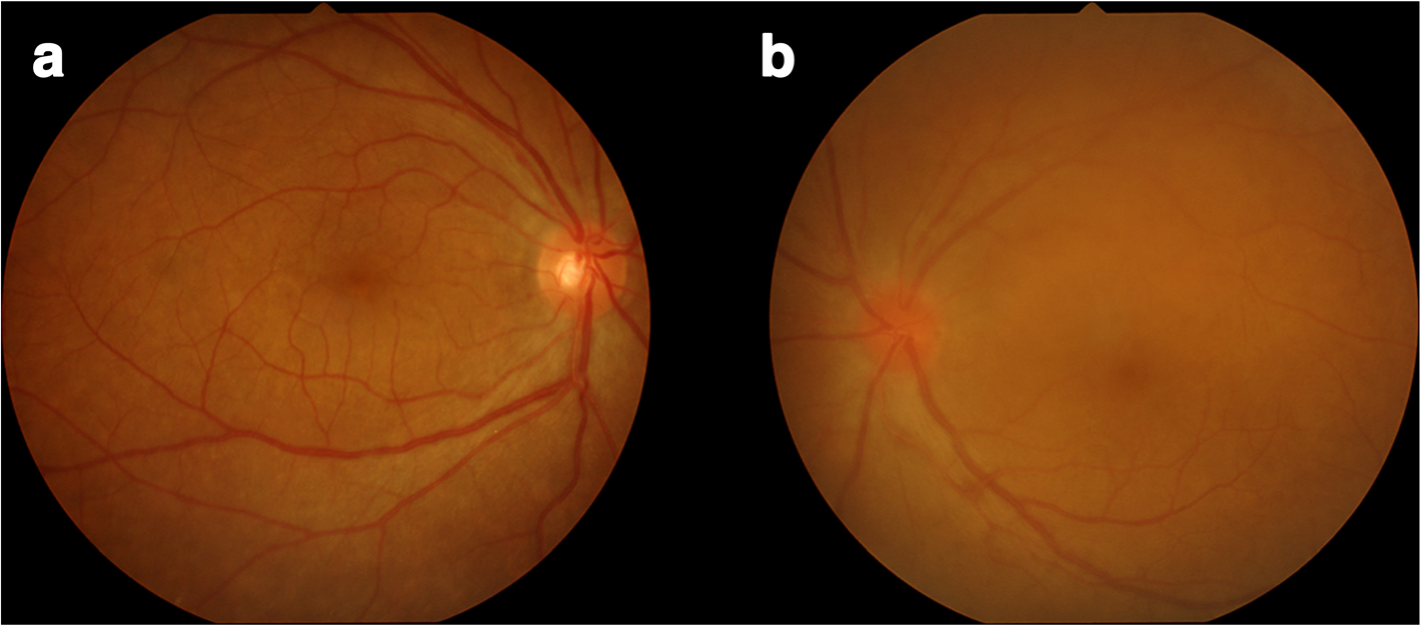
Right (**a**) and left (**b**) fundus. Left optic disc is reddish and swollen

**Fig. 3:**
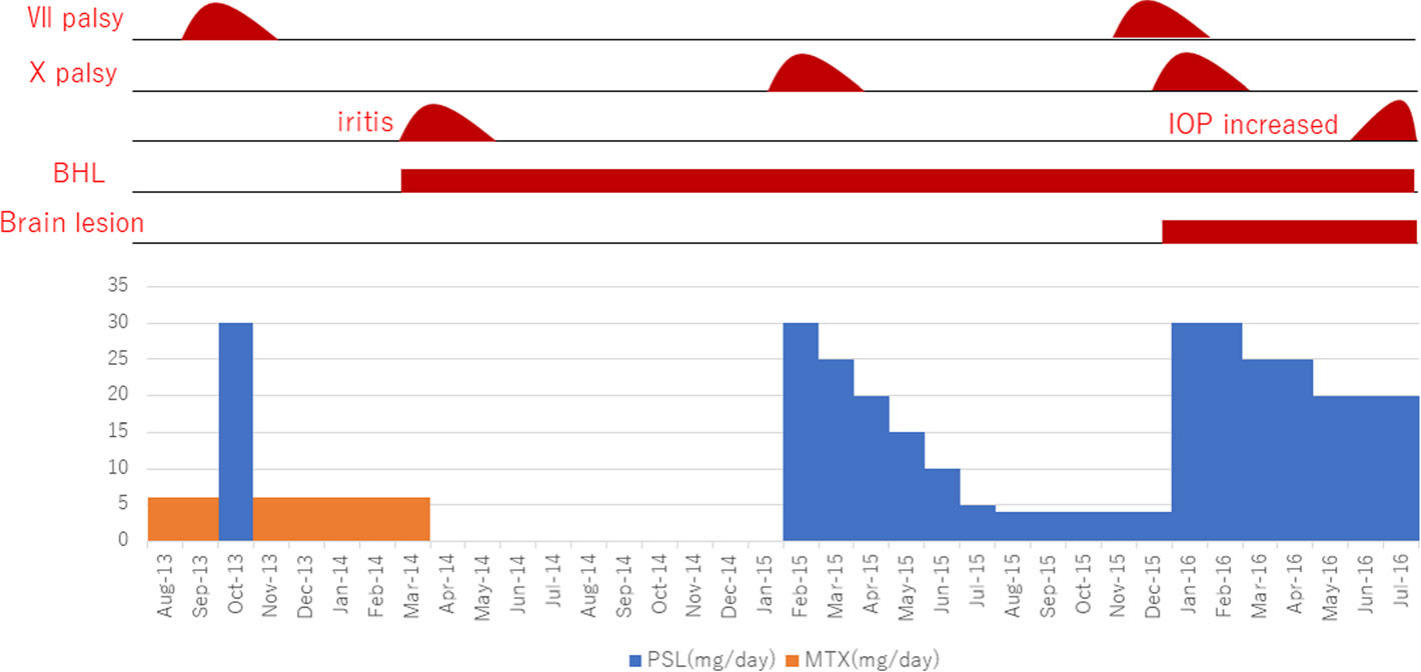
Clinical course. The patient presented with VII nerve palsy and was treated with orally administered prednisolone. However, while prednisolone was tapered off, she developed VII and X nerve palsies, followed by brain parenchyma lesions and increased intraocular pressure in her right eye. *BHL* bilateral hilar lymphadenopathy, *IOP* intraocular pressure, *MTX* methotrexate, *PSL* prednisolone

At the initial visit, her best corrected visual acuity was 20/20 in her right eye and 20/28 in her left eye. The IOP was 40 mmHg in her right and 14 mmHg in her left eye. There was no inflammation in the anterior chamber, although massive, diffuse vitreous opacities were observed in both eyes (Fig. 2). Retinal periphlebitis and macular edema were detected using fluorescence angiography. In July 2016, she underwent trabeculectomy of her right eye, following which the right eye IOP decreased to 10 mmHg. No postoperative inflammation was observed. As a result, 20 mg of prednisolone/day was continued, and she experienced no worsening of brain parenchyma lesions or recurrence of cranial nerve palsy to date. The clinical course is presented in Fig. 3.

## Literature review

**Fig. 4:**
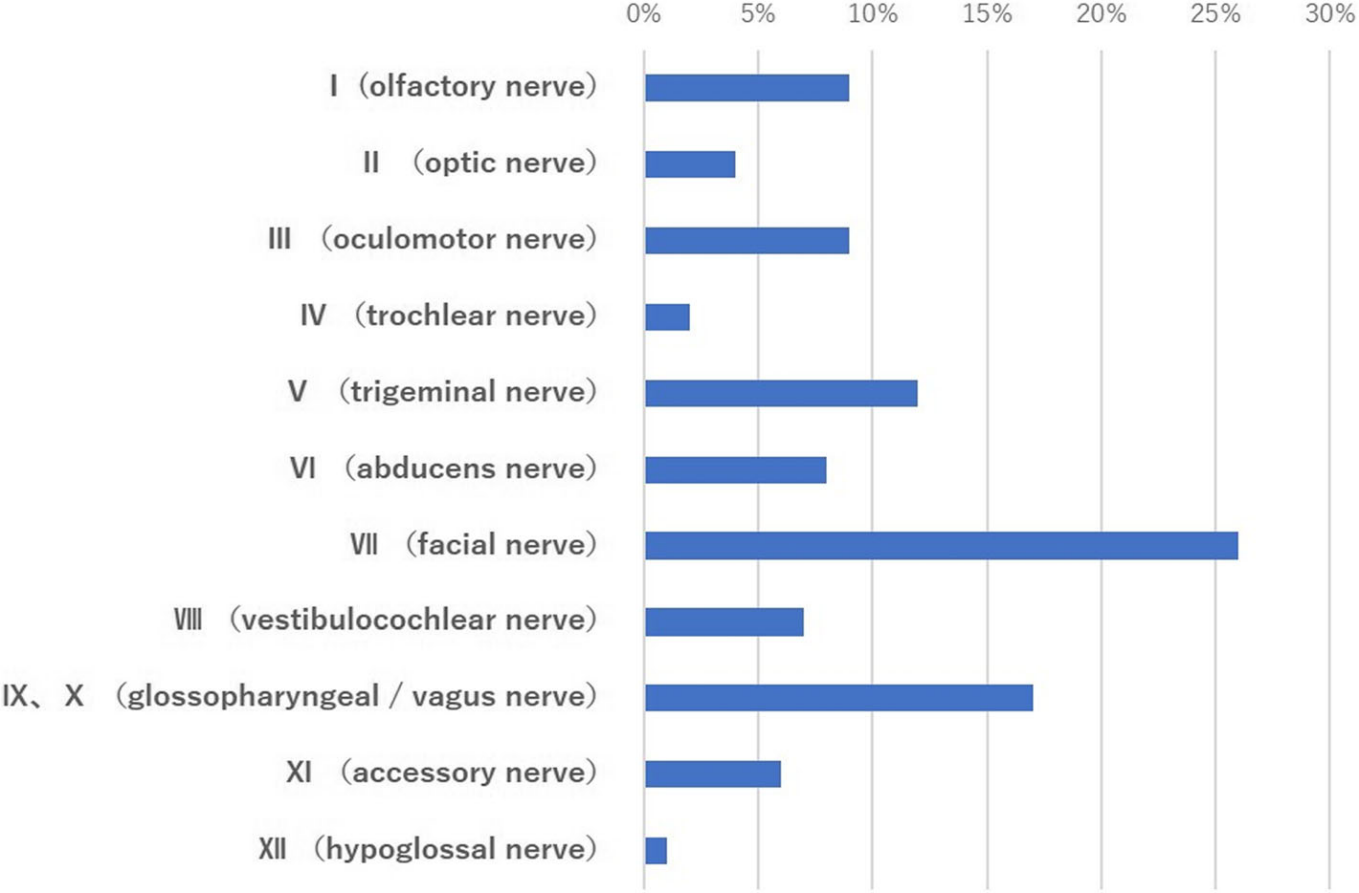
Nerve palsy frequency among 64 Japanese patients with neurosarcoidosis with multiple cranial neuropathies during 1982–2016. The most affected cranial nerve was the facial (VII) nerve (*N* = 47, 73.4%), followed by the glossopharyngeal/vagus (IX/X) nerves (*N* = 31, 48.4%)

We reviewed 64 case reports regarding multiple cranial nerve palsies associated with sarcoidosis written in the Japanese language between January 1982 and December 2016. We searched *Igaku Chuo Zasshi* (Japan’s largest medical literature database) using the terms “multiple cranial neuropathies” and “neurosarcoidosis.” The most commonly affected cranial nerve was nerve VII (47 cases, 73.4%), followed by nerves IX/X (31 cases, 48.4%) (Fig. 4). The proportion of patients with cranial nerve palsies affecting multiple nerves were as follows: two nerves, 40.6% (*N* = 26); three nerves, 23.4% (*N* = 15); four nerves, 18.8% (*N* = 12) (Table 1). Almost all patients (57/58 cases, 98.3%) received systemic steroid therapy. Orally administered prednisolone and pulsed steroid therapy were performed in 38/57 (66.7%) and 19/57 cases (33.3%), respectively. Total or partial remission was achieved in 55/57 cases (96.5%).

**Table 1:** Proportion of patients with cranial nerve palsies affecting multiple nerves

Number of cranial nerves affected	Number of patients	Proportion of patients (%)
2 nerves	26	40.6
3 nerves	15	23.4
4 nerves	12	18.8
5 nerves	3	4.7
6 nerves	5	7.8
7 nerves	2	3.1
8 nerves	1	1.6
Total	64	100

## Discussion and conclusions

Our patient developed palsies of cranial nerves VII and X, which are the most commonly affected nerves associated with sarcoidosis in Japan. Gascón-Bayarri *et al.* [4] reported that 17 of 30 patients with neurosarcoidosis presented with cranial nerve palsy (56.7%). Among these, 14 (82.4%) presented with nerve VII palsy, while rates of nerve VIII (23.5%) and X (17.6%) palsy were much lower. However, one meta-analysis reported that cranial nerves VII and II are the most affected nerves [3]. However, accumulating evidence indicates that cranial nerve VII is among the most affected in both Japanese and non-Japanese patients [3, 4].

Brain parenchyma lesions, which present as headaches and seizures, represent the second-most common manifestation of neurosarcoidosis [2, 4]. In our patient, brain parenchyma lesions were not associated with neurological symptoms, suggesting that these lesions were too small to result in compression of brain tissue. Although highly probable, a definitive diagnosis could not be achieved because a brain biopsy was not performed. However, careful follow-up is necessary, as neurological symptoms may appear if the size of the lesioned area increases.

Previous studies have reported that prednisolone is effective in treating neurosarcoidosis [2, 3, 6]. Our review indicated that 66.7% and 33.3% of Japanese patients received orally administered prednisolone and pulsed steroid therapy, respectively. Furthermore, total remission was achieved in 96.5% of patients. In a recent case series, Ungprasert *et al.* [7] discussed the treatment of five patients with cranial nerve palsy, four of whom received orally administered prednisolone, while one received pulsed steroid therapy. Good response and no recurrence of cranial nerve palsy were observed in four patients (80%). These findings indicate that prednisolone is effective in treating this disease (that is, peripheral nervous system). However, patients with sarcoidosis affecting the CNS exhibit a poorer prognosis than those with sarcoidosis affecting the peripheral nervous system only [2]. Recent studies have indicated that anti-tumor necrosis factor alpha (TNF-α) agents and immunosuppressive agents, such as methotrexate, azathioprine, and cyclosporine, are effective in treating neurosarcoidosis, particularly within the CNS [3, 6–9]. However, the rates of recurrence and infection remain high in patients treated with anti-TNF-α agents [8] and are not associated with overall changes in mortality [3]. Our patient received orally administered prednisolone, following which she experienced recurrence of nerve VII and X palsies and developed brain parenchyma lesions. Thus, it may be necessary to add immunosuppressive agents or TNF-α agents during recurrence or development of additional cranial nerve palsies, or when asymptomatic brain lesions become symptomatic.

In summary, we presented a patient with sarcoidosis with uveitis and multiple neurological findings, such as multiple cranial nerve palsies and asymptomatic brain parenchyma lesions. In the literature, it is commonly reported that patients with multiple cranial nerve palsies associated with sarcoidosis respond well to orally administered steroid therapy. However, careful follow-up is necessary for patients with neurosarcoidosis due to the potential for relapse and aggravation of neuropathy.

## Abbreviations

CNS: Central nervous system
IOP: Intraocular pressure
TNF-α: Tumor necrosis factor alpha
